# Characteristics and outcomes of cancer patients admitted to intensive care units in cancer specialized hospitals in China

**DOI:** 10.1007/s00432-024-05727-0

**Published:** 2024-04-20

**Authors:** Wensheng Liu, Dongmin Zhou, Li Zhang, Mingguang Huang, Rongxi Quan, Rui Xia, Yong Ye, Guoxing Zhang, Zhuping Shen, the Cancer Critical Care Medicine Committee of the Chinese Anti-Cancer Association

**Affiliations:** 1 Department of Intensive Care Unit, Hangzhou Institute of Medicine (HIM), Chinese Academy of Sciences, Zhejiang Cancer Hospital, No. 1 East Banshan Road, Hangzhou, 310022 China; 2 Department of Intensive Care Unit, Henan Cancer Hospital, Zhengzhou, China; 3 Department of Intensive Care Unit, Hubei Cancer Hospital, Wuhan, China; 4 Department of Intensive Care Unit, Shanxi Province Cancer Hospital, Taiyuan, China; 5 Department of Intensive Care Unit, Cancer Hospital of Xinjiang Uygur Autonomous Region, Urumqi, China; 6 Department of Intensive Care Unit, Tianjin Medical University Cancer Institute and Hospital, Tianjin, China; 7 Department of Intensive Care Unit, Fujian Cancer Hospital and Fujian Medical University Cancer Hospital, Fuzhou, China; 8 Department of Intensive Care Unit, Gaoxin District of Jilin Cancer Hospital, Changchun, China

**Keywords:** Cancer specialized hospital, Intensive care unit, Cancer patients, Characteristics and outcomes

## Abstract

**Purpose:**

Standard intensive care unit (ICU) admission policies and treatment strategies for patients with cancer are still lacking. To depict the current status of admission, characteristics, and outcomes of patients with cancer in the ICU.

**Methods:**

A multicenter cross-sectional study was performed from May 10, 2021 to July 10, 2021, in the ICU departments of 37 cancer-specialized hospitals in China. Clinical records of all admitted patients aged ≥ 14 years and ICU duration > 24 h with complete data were included. Demographic information, clinical history, severity score at admission, ICU critical condition diagnosis and treatment, ICU and in-hospital outcomes and 90 days survival were also collected. A total of 1455 patients were admitted and stayed for longer than 24 h. The most common primary cancer diagnoses included lung, colorectal, esophageal, and gastric cancer.

**Results:**

Patients with lung cancer were admitted more often because of worsening complications that occurred in the clinical ward. However, other cancer patients may be more likely to be admitted to the ICU because of postoperative care. ICU-admitted patients with lung or esophageal cancer tended to have more ICU complications. Patients with lung cancer had a poor overall survival prognosis, whereas patients with colorectal cancer appeared to benefit the most according to 90 days mortality rates.

**Conclusion:**

Patients with lung cancer require more ICU care due to critical complications and the overall survival prognosis is poor. Colorectal cancer may benefit more from ICU management. This information may be considered in ICU admission and treatment strategies.

**Supplementary Information:**

The online version contains supplementary material available at 10.1007/s00432-024-05727-0.

## Introduction

Developments in cancer screening and treatment have significantly increased the survival of cancer patients in recent years. Owing to complications or treatment-associated side effects, cancer patients may need to be admitted to the intensive care unit (ICU) to improve survival (Koutsoukou 2017; Martos-Benítez et al. 2020). Admission of cancer patients to the ICU is not encouraged due to low survival rates; however, the progression of cancer treatment and ICU management has significantly increased the life span of cancer patients, and more patients may benefit from ICU visits (Kiehl et al. 2018; Martos-Benítez et al. 2020; Shimabukuro-Vornhagen 2021). It has been estimated that 5% of cancer patients may experience ICU admission (Puxty et al. 2015), and cancer patients account for 15% of ICU patients (Azoulay et al. 2017).

However, the decision for ICU admission is often challenging (Valley et al. 2023). In balancing potential medical outcomes, individual rights and desires, and economic constraints and availability of resources, it is important to form instructive admission policies based on the predicted benefits for the patients (Toffart et al. 2023). However, well accepted and applicable ICU admission strategies for cancer patients are still lacking as criteria suggested by previous studies were often very personalized (Kostakou et al. 2014; Lecuyer et al. 2007; Soares et al. 2004). In addition, indicators facilitating the identification of patients who are more likely to benefit from ICU management are often very complex, with limited prognostic values (Staudinger et al. 2000; Thiéry et al. 2005). Therefore, there is a demand for instructive ICU admission policies and treatment strategies for cancer patients (Koutsoukou 2017).

A clear picture of the current admission status and outcomes of cancer patients in the ICU is of great clinical importance because it not only helps to identify the existing problems in the current admission policy but also provides clues to patients who are more likely to benefit from ICU management. Many published studies have analyzed the clinical features, outcomes, and predictors of patients with cancer who have undergone ICU treatments (Epstein et al. 2020; Kemoun et al. 2023; Xu et al. 2023; Zampieri et al. 2021). However, they are often more concentrated in specific malignancy fields. In addition, few studies and data are available from China, which has a significant population of cancer patients. In addition, ICU departments in cancer-specialized hospitals are common in China, which may require specific organization concerning ICU admission and management policies.

Therefore, a multicenter study of 37 ICU departments of cancer-specialized hospitals was performed and the results are summarized here to highlight the patients’ characteristics and outcomes.

## Material and methods

### Study design and population

A multicenter cross-sectional study was performed from May 10, 2021 to July 10, 2021 at the ICU departments of 37 specialized cancer hospitals in China, with the aim of depicting the admission status and characteristics of critically ill patients. The clinical records of all admitted patients in the ICUs of participating centers were screened and considered eligible if the following information was complete: reasons for admission to the ICU and underlying medical history, severity evaluation at the time of ICU admission, incidence of sepsis, acute respiratory distress syndrome (ARDS), acute kidney injury (AKI), treatment of intensive care medicine-related diagnosis and treatment regimen, in-hospital outcomes, and 90-day survival at follow-up. Patients aged < 14 years and those with an ICU stay < 24 h were excluded.

A detailed list of the participating centers is included in the supplemental information. This study was approved by Ethic committee of Tianjin Medical University Cancer Institute and Hospital, and informed consent was obtained from all the participants and the legal guardians of the minors.

### Data collection

The following data were extracted from the clinical records of the admitted patients. (1) General demographic information including age, sex, and BMI. (2) Clinical history, including patient transfer source, primary diagnosis, and cancer treatment history. (3) Evaluation results at admission, including SOFA and APACHE II scores and laboratory examination results. (4) Critical ICU diagnosis, including sepsis and septic shock, ARDS, respiratory failure, and AKI. (5) Treatment of intensive care, including anti-infection medication, respiratory support, renal replacement therapy, sedation, and nutritional regimens. (6) Outcomes included the occurrence of delirium, ICU duration, ICU death, and in-hospital death. In addition, the 90 days survival information was collected during follow-up.

### Statistical analysis

The Shapiro–Wilk test was performed to check the normality of the quantitative data distribution. Due to the skewed distribution, the median and quartile ranges (P25, P75) were used to describe quantitative data. Categorical data were presented as frequencies (n) and percentages (%). Quantitative data were compared among the groups using the Kruskal–Wallis H test. Categorical data were compared between groups using either the χ^2^ test or Fisher’s exact test. Software R 4.3.1 (R Foundation for Statistical Computing, Vienna, Austria) was used for statistical analysis, and all tests were two-sided with a test level of α = 0.05.

## Results

### Patients and baseline characteristics

A total of 1455 patients aged ≥ 14 years were admitted and stayed longer than 24 h in 37 ICU departments. The most common primary cancer diagnoses included lung (269, 18.5%), colorectal (189, 13.0%), esophageal (176, 12.1%), and gastric (113, 7.8%) cancer. In addition, 62 patients (4.3%) were diagnosed with various hematological tumors. The number of admitted patients with other cancer types (nasopharyngeal, pancreatic, gallbladder, liver, ovarian, cervical, thyroid, melanoma, brain, prostate, breast, kidney, etc.) was less than 50.

As shown in Table 1, the admitted patients had an average age of 65 years and a significantly higher percentage of males (61.4%). Differences in various baseline characteristics among the patients with different primary cancer diagnoses were detected. It may be worth noticing that patients with lung cancers had higher APACHE II scores compared to other types of cancer. A significantly lower percentage (27.5%) of lung cancer patients were admitted through planned transfers and most lung cancer patients (65.8%) were transferred from the clinical ward. Additionally, fewer patients with lung cancer (38.7%) underwent elective surgery. For the other three major cancer types (colorectal, esophageal, and gastric), approximately half (49.7%, 43.8%, and 47.8%, respectively) were admitted through planned transfer and more (69.3%, 74.4%, and 62.8%, respectively) underwent elective surgery.

**Table 1:** Baseline characteristics of admitted patients in ICUs of cancer specialized hospitals in China

	All (*n* = 1455)	Lung (*n* = 269)	Colorectal (*n* = 189)	Esophageal (*n* = 176)	Gastric (*n* = 113)	Others (*n* = 708)	*P* value
Demographic							
Age (year)	65.0 (56.0, 72.0)	65.0 (58.0, 71.0)	69.0 (60.0, 76.0)	67.0 (61.0, 72.0)	67.0 (58.0, 75.0)	62.0 (53.0, 70.0)	< 0.001
Gender							< 0.001
Female	562 (38.6)	77 (28.6)	58 (30.7)	45 (25.6)	41 (36.3)	341 (48.2)	
Male	893 (61.4)	192 (71.4)	131 (69.3)	131 (74.4)	72 (63.7)	367 (51.8)	
BMI (kg/m2)	22.4 (20.0, 24.8)	22.2 (20.3, 24.7)	22.5 (19.8, 25.1)	22.4 (19.5, 24.3)	21.6 (19.2, 24.2)	22.5 (20.2, 24.9)	0.114
Admission evaluation							
SOFA	3.0 (2.0, 6.0)	3.0 (2.0, 6.0)	3.0 (2.0, 5.0)	3.0 (2.0, 5.0)	3.0 (1.0, 6.0)	3.0 (2.0, 6.0)	0.182
APACHE II	12.0 (8.0, 17.0)	***13.0 (9.0, 19.0)***	11.0 (8.0, 15.0)	11.5 (8.0, 16.0)	11.0 (7.0, 15.0)	12.0 (8.0, 18.0)	***0.003***
Source of transfer							** < *0.001***
Operating room	594 (40.8)	68 (25.3)	119 (63.0)	75 (42.6)	66 (58.4)	266 (37.6)	
Emergency department	63 (4.3)	16 (5.9)	7 (3.7)	3 (1.7)	5 (4.4)	32 (4.5)	
Clinical ward	781 (53.7)	***177 (65.8)***	***61 (32.3)***	***98 (55.7)***	***42 (37.2)***	403 (56.9)	
Other hospitals	17 (1.2)	8 (3.0)	2 (1.1)	0 (0.0)	0 (0.0)	7 (1.0)	
Planned transfer							** < *0.001***
No	895 (61.5)	***195 (72.5)***	***95 (50.3)***	***99 (56.2)***	***59 (52.2)***	447 (63.1)	
Yes	560 (38.5)	74 (27.5)	94 (49.7)	77 (43.8)	54 (47.8)	261 (36.9)	
Elective or emergency surgery							** < *0.001***
No surgery	535 (36.8)	161 (59.9)	33 (17.5)	41 (23.3)	26 (23.0)	274 (38.7)	
Elective surgery	819 (56.3)	***104 (38.7)***	***131 (69.3)***	***131 (74.4)***	***71 (62.8)***	382 (54.0)	
Emergency surgery	101 (6.9)	4 (1.5)	25 (13.2)	4 (2.3)	16 (14.2)	52 (7.3)	

These results indicate that lung cancer patients were more likely to require ICU care and were more often admitted due to worsening complications that occurred in the clinical ward. However, other cancer patients may be more likely to be admitted to the ICU because of postoperative care.

### ICU diagnosis and medical treatments

The ICU complication diagnoses and medical treatments are summarized in Table 2. During ICU stay, 918 (63.1%) patients were diagnosed with sepsis. Patients with lung cancer (73.2%) or esophageal cancer (71.6%) had a significantly higher percentage of sepsis than those with colorectal cancer (55.0%) and gastric cancer (52.2%). Respiratory failure and ARDS were diagnosed in 550 (37.8%) and 198 (13.6%) patients, respectively. AKI occurred in 172 (11.8%) patients. Patients with lung cancer or esophageal cancer had significantly higher risks of respiratory failure, ARDS, and AKI than those with colorectal and gastric cancers. Shock was diagnosed in 404 patients (27.8%) and no significant percentage differences were detected among the most common cancer types.

**Table 2:** Diagnosis of ICU complications and ICU treatments of cancer patients

	All (*n* = 1455)	Lung (*n* = 269)	Colorectal (*n* = 189)	Esophageal (*n* = 176)	Gastric (*n* = 113)	Others (*n* = 708)	*P* value
ICU diagnosis							
Sepsis							< 0.001
No	537 (36.9)	72 (26.8)	85 (45.0)	50 (28.4)	54 (47.8)	276 (39.0)	
Yes	918 (63.1)	197 (73.2)	104 (55.0)	126 (71.6)	59 (52.2)	432 (61.0)	
ARDS							< 0.001
No	1257 (86.4)	213 (79.2)	172 (91.0)	138 (78.4)	102 (90.3)	632 (89.3)	
Yes	198 (13.6)	56 (20.8)	17 (9.0)	38 (21.6)	11 (9.7)	76 (10.7)	
Respiratory failure							< 0.001
No	905 (62.2)	129 (48.0)	141 (74.6)	78 (44.3)	83 (73.5)	474 (66.9)	
Yes	550 (37.8)	140 (52.0)	48 (25.4)	98 (55.7)	30 (26.5)	234 (33.1)	
AKI							0.002
No	1283 (88.2)	234 (87.0)	171 (90.5)	170 (96.6)	100 (88.5)	608 (85.9)	
Yes	172 (11.8)	35 (13.0)	18 (9.5)	6 (3.4)	13 (11.5)	100 (14.1)	
Shock							0.958
No	1051 (72.2)	196 (72.9)	134 (70.9)	129 (73.3)	79 (69.9)	513 (72.5)	
Yes	404 (27.8)	73 (27.1)	55 (29.1)	47 (26.7)	34 (30.1)	195 (27.5)	
ICU treatment							
Anti-infection							< 0.001
None	496 (34.1)	69 (25.7)	80 (42.3)	41 (23.3)	53 (46.9)	253 (35.7)	
Antifugal and antibacteria	204 (14.0)	65 (24.2)	10 (5.3)	20 (11.4)	11 (9.7)	98 (13.8)	
Antibacteria	755 (51.9)	135 (50.2)	99 (52.4)	115 (65.3)	49 (43.4)	357 (50.4)	
Mechanical Ventilation							0.003
No	816 (56.1)	144 (53.5)	113 (59.8)	77 (43.8)	72 (63.7)	410 (57.9)	
Yes	639 (43.9)	125 (46.5)	76 (40.2)	99 (56.2)	41 (36.3)	298 (42.1)	
Conventional oxygen therapy							< 0.001
No	231 (15.9)	76 (28.3)	15 (7.9)	32 (18.2)	8 (7.1)	100 (14.1)	
Yes	1224 (84.1)	193 (71.7)	174 (92.1)	144 (81.8)	105 (92.9)	608 (85.9)	
Sedation treatment							< 0.001
No	1030 (70.8)	172 (63.9)	141 (74.6)	95 (54.0)	87 (77.0)	535 (75.6)	
Yes	425 (29.2)	97 (36.1)	48 (25.4)	81 (46.0)	26 (23.0)	173 (24.4)	

As for the ICU treatment administered, anti-infection treatments were applied in 65.1% of the patients, with significantly lower percentages of gastric (53.1%) and colorectal (57.7%) cancer patients than in lung (74.3%) and esophageal (76.7%) cancer patients. Antifungal medications were administered to 14% of the patients, with significantly higher usage (24.2%) in lung cancer patients and significantly lower usage (5.3%) in colorectal cancer patients. Mechanical ventilation was applied in 43.9% of the patients, with a higher percentage (56.2%) of patients with esophageal cancer. Sedation treatments were administered to 29.2% of patients, with significantly higher percentages for patients receiving lung (36.1%) and esophageal (46.0%) cancer treatments.

These results indicate that ICU-admitted patients with lung or esophageal cancer tended to have more ICU complications and were more likely to receive more intense treatment regimens.

### Survival outcomes

A total of 85 (5.8%) and 104 (7.1%) patients died in the ICU or before hospital discharge, respectively. As shown in Table 3, patients with lung cancer had significantly higher ICU (11.9%) and in-hospital (15.2%) mortality rates than those with other types of cancer. The mortality rate further increased during the short period after discharge from the hospital, with an overall in-hospital death rate of 7.1%, which increased to 27.8% for the 90 days mortality rate. Lung cancer patients had the highest 90 days mortality rate (45.4%), whereas colorectal cancer patients had the lowest 90 days mortality rate (12.2%). For esophageal and gastric cancer patients, the mortality rates increased from in-hospital death of 4.0% and 5.3% to 90 days death by 22.7% and 27.4%, respectively.

**Table 3:** Survival outcomes of cancer patients admitted into the ICU

	All (*n* = 1455)	Lung (*n* = 269)	Colorectal (*n* = 189)	Esophageal (*n* = 176)	Gastric (*n* = 113)	Others (*n* = 708)	*P* value
Survival outcomes							
ICU death							< 0.001
No	1370 (94.2)	237 (88.1)	185 (97.9)	170 (96.6)	108 (95.6)	670 (94.6)	
Yes	85 (5.8)	32 (11.9)	4 (2.1)	6 (3.4)	5 (4.4)	38 (5.4)	
In-hospital death							< 0.001
No	1351 (92.9)	228 (84.8)	182 (96.3)	169 (96.0)	107 (94.7)	665 (93.9)	
Yes	104 (7.1)	41 (15.2)	7 (3.7)	7 (4.0)	6 (5.3)	43 (6.1)	
90 days death							< 0.001
No	1050 (72.2)	147 (54.6)	166 (87.8)	136 (77.3)	82 (72.6)	519 (73.3)	
Yes	405 (27.8)	122 (45.4)	23 (12.2)	40 (22.7)	31 (27.4)	189 (26.7)	

These results indicate that patients with cancer who received ICU care had significant short-term mortality risks. Nearly half of ICU-admitted patients with lung cancer had a short-term mortality prognosis. Patients with colorectal cancer are most likely to benefit from ICU management. This information may be utilized in future adjustments of admission and treatment strategies.

## Discussion

The results of the current study show the ICUs of cancer-specialized hospitals in China. The most commonly admitted patients had lung, colorectal, esophageal, and gastric cancer. Unplanned transfer from clinical wards is more likely to occur in lung cancer patients, while other types of cancer patients are more often transferred from operating rooms because of postoperative care needs. Sepsis and intensive respiratory complications are more likely to occur in patients with lung or esophageal cancers. Nearly half of patients with lung cancer have a short-term mortality prognosis. In contrast, ICU-admitted patients with colorectal cancer had the best survival prognosis.

The most commonly admitted patients in cancer ICUs in this study were lung, colorectal, esophageal, and gastric cancers. Epidemiological studies have identified that these are also the most common cancer types in China (Fan et al. 2022; Xia et al. 2022) and among the most common cancer types in the world (Siegel et al. 2023). In addition, it has been suggested that lung cancer patients are at a higher risk of ICU admission and may present in 27% of all patients with solid cancers (Park et al. 2021). These results were consistent with our findings. In addition to solid tumors, slightly less than 5% of the homological malignancies were found in ICU-admitted patients in this study. It has been suggested that patients with homological malignancies are more likely to experience ICU-related emergencies (Velmurugan et al. 2022), and previous studies have found that 10% (Soares et al. 2016; Zampieri et al. 2021) to 21% (Na et al. 2018) of ICU admitted cancer patients have homological malignancies. Homological malignancies were more common in children and patients younger than 14 years were excluded from the current study. This may explain the differences and suggests that information on ICU patients with homological malignancies needs to be further completed in future data collection.

The common reasons for ICU admission of cancer patients include oncology- or treatment-related complications, mainly acute respiratory failure (ARF), infection and sepsis, cardiac complications, neurological disorders, and acute kidney injury. In addition, patients may be transferred due to postoperative care (Biskup et al. 2017). It has been suggested that patients with lung cancer had a higher risk of ARF as well as bacterial infection (Cupp et al. 2018; Williams et al. 2023). It is not surprising since the lung is the main respiratory organ, more accessible to environmental pathogens, as well as plays a critical role in the immune system (Kumar 2020). Also, treatments for lung cancer may involve fewer surgical procedures than the cancers of digestive system organs. This may explain why there were more patients with lung cancer transferred from the clinical ward instead of the operating room and why they were more likely to be an unplanned transfer.

Planned transfer and transfer after elective surgery usually aim for postoperative care. In such cases, it would be expected that serious, life-limiting complications are unlikely to occurred. However, when the rates of planned and unplanned transfers after elective and emergency surgeries, as well as the diagnosis of sepsis, as a representative of severe complications, were analyzed, the results (Online Resource 1) showed that for patients transferred into ICUs after elective surgeries, 65.4% were planned transfer, among them 32.5% were diagnosed with sepsis during ICU management. Unplanned transfer accounted for 34.6% of total ICU admission after elective surgeries, among them 72.9% were reported with sepsis during ICU management. ICU admission after emergency surgeries were mainly unplanned (93.4%), of which 80.8% were reported with sepsis during ICU management. These results indicated that although planned ICU transfer after elective surgeries were usually only for postoperative care, there still exist the risks of severe complications in these patients. The general principle of postoperative care for elective operation was that patients are routinely admitted to the PACU or postoperative recovery room for observation after surgery. Only patients considered with high risk due to elder ages, complicated underlying diseases, and large surgical scope were subjected to ICU care for subsequent monitoring and treatment. The appeared relatively high rates of ICU applications therefore may be explained by the weaker baseline health conditions of patients admitted to ICU. It should also be pointed out that the study was carried out in 37 hospitals, with more than 250,000 annual operations performed. Although the exact number need to be confirmed, the percentage of patients admitted into ICUs after elective operation were actually less than 3%.

During ICU management, the diagnosis of sepsis, ARF, ARDS, and AKI occurred more often in patients with lung cancer and esophageal cancer, suggesting a higher risk of ICU complications in these patients. As mentioned above, the higher risk of severe complications in lung cancer may be explained by the etiology and treatment regimen. Higher risks of infection have also been suggested in esophageal cancer (Kawasaki et al. 2021; Zheng et al. 2022). Higher chances of radiotherapy may also increase the risk of infection and ARF in esophageal cancer (Terrones-Campos et al. 2022).

The results also showed that a significant number of patients died within a short period after hospital discharge. In patients with lung cancer, nearly 50% mortality was observed at 90 days. These results suggest that the overall potential benefits of ICU management should be assessed more carefully during admission. Patients admitted to ICUs in cancer specialized hospitals were often at advanced stages of cancers. These patients were generally with poor prognosis even survived in-hospital treatments. Previous studies in ICU admitted cancer patients have also reported high short-term mortalities. For example, one study reported that ICU treated gastrointestinal cancer patients were reported to have a median survival of only 2.2 months even after hospital discharge (Epstein et al. 2020). Another study showed that even when ICU mortality was only 26%, the 3-month mortality of ICU admitted pancreatic cancer patients was 59% (Kemoun et al. 2023). Large sample sized study from Korea showed that for patients with solid tumor, after ICU discharge, nearly another 20% patients died in hospital (Na et al. 2018). In lung cancer, the population based study from Korea found that mortality in ICU, 60 days and 1 year were 24.4%, 33.8%, and 49.9%, respectively (Park et al. 2021). The overall 90-day mortality in this study were in accordance with the previous findings. However, it should be pointed out, that the low mortality during ICU stay indicated the discharge policy after management of life-threatening conditions may be different for those with severe cancer related prognosis in the participating hospitals.

The study had limitations, the data were collected based on regular ICU clinical procedures and completeness needs to be further improved. For example, cardiovascular complications are among the most common reasons for ICU admission in patients with cancer. However, these data were not analyzed in the current study. In addition, all the data were from cancer-specialized hospitals, while general tertiary hospitals, which represent one of the major treatment resources for cancer in China, were not included. Therefore, the characteristics identified in this study may only show the perspective of ICU-admitted cancer patients in China.

## Conclusion

In conclusion, the ICU admission rates of patients with cancer were proportional to the prevalence of different cancer types. Patients with lung cancer might require more ICU care due to critical complications and their overall survival prognosis is poor. Patients with colorectal cancer appear to benefit the most from ICU management. ICU admission standards may be further optimized according to this information.

### Supplementary Information

Below is the link to the electronic supplementary material.

Supplementary file1 (DOCX 17 KB)

Supplementary file2 (DOCX 27 KB)

## Data Availability

The datasets generated during and/or analyzed during the current study are available from the corresponding author on reasonable request.
