# Supplementary material for: Characteristics and outcomes of cancer patients admitted to intensive care units in cancer specialized hospitals in China
Source: J Cancer Res Clin Oncol. 2024 Apr 20;150(4):205. doi: 10.1007/s00432-024-05727-0 (PMC11032264; doi:10.1007/s00432-024-05727-0)
Supplement: Supplementary file 1 — Supplementary file1 (DOCX 17 KB) [file 432_2024_5727_MOESM1_ESM.docx]

Online Resource 1. Rates of unplanned transfer and sepsis in different surgeries

| Sepsis | 177 (32.5%) | 210 (72.9%) |
| --- | --- | --- |
| Non-sepsis | 368 (67.5%) | 78 (27.1%) |
| Elective Surgery | Planned transfer 545 (65.4%) | Unplanned transfer 288 (34.6%) |
| Emergency Surgery | Planned transfer 7 (6.6%) | Unplanned transfer 99 (93.4%) |
| Non-sepsis | 2 (28.6%) | 19 (19.2%) |
| Sepsis | 5 (71.4%) | 80 (80.8%) |
