# Supplementary material for: Characteristics and outcomes of cancer patients admitted to intensive care units in cancer specialized hospitals in China
Source: J Cancer Res Clin Oncol. 2024 Apr 20;150(4):205. doi: 10.1007/s00432-024-05727-0 (PMC11032264; doi:10.1007/s00432-024-05727-0)
Supplement: Supplementary file 2 — Supplementary file2 (DOCX 27 KB) [file 432_2024_5727_MOESM2_ESM.docx]

# Supplementary Information

Online Resource 1. Rates of unplanned transfer and sepsis in different surgeries

**Table 1. Baseline characteristics of admitted patients in ICUs of cancer specialized hospitals in China.**

|  | All  (n = 1455) | Lung  (n = 269) | Colorectal  (n = 189) | Esophageal  (n = 176) | Gastric  (n = 113) | Others  (n = 708) | P-value |
| --- | --- | --- | --- | --- | --- | --- | --- |
| Demographic |  |  |  |  |  |  |  |
| Age (year) | 65.0 (56.0, 72.0) | 65.0 (58.0, 71.0) | 69.0 (60.0, 76.0) | 67.0 (61.0, 72.0) | 67.0 (58.0, 75.0) | 62.0 (53.0, 70.0) | <0.001 |
| Gender |  |  |  |  |  |  | <0.001 |
| Female | 562 (38.6) | 77 (28.6) | 58 (30.7) | 45 (25.6) | 41 (36.3) | 341 (48.2) |  |
| Male | 893 (61.4) | 192 (71.4) | 131 (69.3) | 131 (74.4) | 72 (63.7) | 367 (51.8) |  |
| BMI (kg/m2) | 22.4 (20.0, 24.8) | 22.2 (20.3, 24.7) | 22.5 (19.8, 25.1) | 22.4 (19.5, 24.3) | 21.6 (19.2, 24.2) | 22.5 (20.2, 24.9) | 0.114 |
| Admission evaluation |  |  |  |  |  |  |  |
| SOFA | 3.0 (2.0, 6.0) | 3.0 (2.0, 6.0) | 3.0 (2.0, 5.0) | 3.0 (2.0, 5.0) | 3.0 (1.0, 6.0) | 3.0 (2.0, 6.0) | 0.182 |
| APACHE II | 12.0 (8.0, 17.0) | ***13.0 (9.0, 19.0)*** | 11.0 (8.0, 15.0) | 11.5 (8.0, 16.0) | 11.0 (7.0, 15.0) | 12.0 (8.0, 18.0) | ***0.003*** |
| Source of transfer |  |  |  |  |  |  | ***<0.001*** |
| Operating room | 594 (40.8) | 68 (25.3) | 119 (63.0) | 75 (42.6) | 66 (58.4) | 266 (37.6) |  |
| Emergency department | 63 (4.3) | 16 (5.9) | 7 (3.7) | 3 (1.7) | 5 (4.4) | 32 (4.5) |  |
| Clinical ward | 781 (53.7) | ***177 (65.8)*** | ***61 (32.3)*** | ***98 (55.7)*** | ***42 (37.2)*** | 403 (56.9) |  |
| Other hospitals | 17 (1.2) | 8 (3.0) | 2 (1.1) | 0 (0.0) | 0 (0.0) | 7 (1.0) |  |
| Planned transfer |  |  |  |  |  |  | ***<0.001*** |
| No | 895 (61.5) | ***195 (72.5)*** | ***95 (50.3)*** | ***99 (56.2)*** | ***59 (52.2)*** | 447 (63.1) |  |
| Yes | 560 (38.5) | 74 (27.5) | 94 (49.7) | 77 (43.8) | 54 (47.8) | 261 (36.9) |  |
| Elective or emergency surgery |  |  |  |  |  |  | ***<0.001*** |
| No surgery | 535 (36.8) | 161 (59.9) | 33 (17.5) | 41 (23.3) | 26 (23.0) | 274 (38.7) |  |
| Elective surgery | 819 (56.3) | ***104 (38.7)*** | ***131 (69.3)*** | ***131 (74.4)*** | ***71 (62.8)*** | 382 (54.0) |  |
| Emergency surgery | 101 (6.9) | 4 (1.5) | 25 (13.2) | 4 (2.3) | 16 (14.2) | 52 (7.3) |  |

**Table 2. Diagnosis of ICU complications and ICU treatments of cancer patients.**

|  | All  (n = 1455) | Lung  (n = 269) | Colorectal  (n = 189) | Esophageal  (n = 176) | Gastric  (n = 113) | Others  (n = 708) | P-value |
| --- | --- | --- | --- | --- | --- | --- | --- |
| ICU diagnosis |  |  |  |  |  |  |  |
| Sepsis |  |  |  |  |  |  | <0.001 |
| No | 537 (36.9) | 72 (26.8) | 85 (45.0) | 50 (28.4) | 54 (47.8) | 276 (39.0) |  |
| Yes | 918 (63.1) | 197 (73.2) | 104 (55.0) | 126 (71.6) | 59 (52.2) | 432 (61.0) |  |
| ARDS |  |  |  |  |  |  | <0.001 |
| No | 1257 (86.4) | 213 (79.2) | 172 (91.0) | 138 (78.4) | 102 (90.3) | 632 (89.3) |  |
| Yes | 198 (13.6) | 56 (20.8) | 17 (9.0) | 38 (21.6) | 11 (9.7) | 76 (10.7) |  |
| Respiratory failure |  |  |  |  |  |  | <0.001 |
| No | 905 (62.2) | 129 (48.0) | 141 (74.6) | 78 (44.3) | 83 (73.5) | 474 (66.9) |  |
| Yes | 550 (37.8) | 140 (52.0) | 48 (25.4) | 98 (55.7) | 30 (26.5) | 234 (33.1) |  |
| AKI |  |  |  |  |  |  | 0.002 |
| No | 1283 (88.2) | 234 (87.0) | 171 (90.5) | 170 (96.6) | 100 (88.5) | 608 (85.9) |  |
| Yes | 172 (11.8) | 35 (13.0) | 18 (9.5) | 6 (3.4) | 13 (11.5) | 100 (14.1) |  |
| Shock |  |  |  |  |  |  | 0.958 |
| No | 1051 (72.2) | 196 (72.9) | 134 (70.9) | 129 (73.3) | 79 (69.9) | 513 (72.5) |  |
| Yes | 404 (27.8) | 73 (27.1) | 55 (29.1) | 47 (26.7) | 34 (30.1) | 195 (27.5) |  |
| ICU treatment |  |  |  |  |  |  |  |
| Anti-infection |  |  |  |  |  |  | <0.001 |
| None | 496 (34.1) | 69 (25.7) | 80 (42.3) | 41 (23.3) | 53 (46.9) | 253 (35.7) |  |
| Antifugal and antibacteria | 204 (14.0) | 65 (24.2) | 10 (5.3) | 20 (11.4) | 11 (9.7) | 98 (13.8) |  |
| Antibacteria | 755 (51.9) | 135 (50.2) | 99 (52.4) | 115 (65.3) | 49 (43.4) | 357 (50.4) |  |
| Mechanical Ventilation |  |  |  |  |  |  | 0.003 |
| No | 816 (56.1) | 144 (53.5) | 113 (59.8) | 77 (43.8) | 72 (63.7) | 410 (57.9) |  |
| Yes | 639 (43.9) | 125 (46.5) | 76 (40.2) | 99 (56.2) | 41 (36.3) | 298 (42.1) |  |
| Conventional oxygen therapy |  |  |  |  |  |  | <0.001 |
| No | 231 (15.9) | 76 (28.3) | 15 (7.9) | 32 (18.2) | 8 (7.1) | 100 (14.1) |  |
| Yes | 1224 (84.1) | 193 (71.7) | 174 (92.1) | 144 (81.8) | 105 (92.9) | 608 (85.9) |  |
| Sedation treatment |  |  |  |  |  |  | <0.001 |
| No | 1030 (70.8) | 172 (63.9) | 141 (74.6) | 95 (54.0) | 87 (77.0) | 535 (75.6) |  |
| Yes | 425 (29.2) | 97 (36.1) | 48 (25.4) | 81 (46.0) | 26 (23.0) | 173 (24.4) |  |

**Table 3. Survival outcomes of cancer patients admitted into the ICU.**

|  | All  (n = 1455) | Lung  (n = 269) | Colorectal  (n = 189) | Esophageal  (n = 176) | Gastric  (n = 113) | Others  (n = 708) | P-value |
| --- | --- | --- | --- | --- | --- | --- | --- |
| Survival outcomes |  |  |  |  |  |  |  |
| ICU death |  |  |  |  |  |  | <0.001 |
| No | 1370 (94.2) | 237 (88.1) | 185 (97.9) | 170 (96.6) | 108 (95.6) | 670 (94.6) |  |
| Yes | 85 (5.8) | 32 (11.9) | 4 (2.1) | 6 (3.4) | 5 (4.4) | 38 (5.4) |  |
| In-hospital death |  |  |  |  |  |  | <0.001 |
| No | 1351 (92.9) | 228 (84.8) | 182 (96.3) | 169 (96.0) | 107 (94.7) | 665 (93.9) |  |
| Yes | 104 (7.1) | 41 (15.2) | 7 (3.7) | 7 (4.0) | 6 (5.3) | 43 (6.1) |  |
| 90 days death |  |  |  |  |  |  | <0.001 |
| No | 1050 (72.2) | 147 (54.6) | 166 (87.8) | 136 (77.3) | 82 (72.6) | 519 (73.3) |  |
| Yes | 405 (27.8) | 122 (45.4) | 23 (12.2) | 40 (22.7) | 31 (27.4) | 189 (26.7) |  |
